# RETRACTION: A Meta‐Analysis of Clinical Studies of Moxibustion for Pressure Ulcer Healing

**DOI:** 10.1111/iwj.70582

**Published:** 2025-04-18

## Abstract

**RETRACTION:** YuW. , ShiB. , HeQ. , and ZhangS. , “A Meta‐Analysis of Clinical Studies of Moxibustion for Pressure Ulcer Healing,” International Wound Journal 21, no. 2 (2024): e14686, 10.1111/iwj.14686.PMC1083113342052949

The above article, published online on 31 January 2024, in Wiley Online Library (http://onlinelibrary.wiley.com/), has been retracted by agreement between the journal Editor in Chief, Professor Keith Harding; and John Wiley & Sons Ltd. Following an investigation by the publisher, all parties have concluded that this article was accepted solely on the basis of a compromised peer review process. The editors have therefore decided to retract the article. The authors did not respond to our notice regarding the retraction.

**RETRACTION:** W. Yu , B. Shi , Q. He , and S. Zhang , “A Meta‐Analysis of Clinical Studies of Moxibustion for Pressure Ulcer Healing,” International Wound Journal 21, no. 2 (2024): e14686, 10.1111/iwj.14686.PMC1083113342052949

The above article, published online on 31 January 2024, in Wiley Online Library (http://onlinelibrary.wiley.com/), has been retracted by agreement between the journal Editor in Chief, Professor Keith Harding; and John Wiley & Sons Ltd. Following an investigation by the publisher, all parties have concluded that this article was accepted solely on the basis of a compromised peer review process. The editors have therefore decided to retract the article. The authors did not respond to our notice regarding the retraction.

